# Notes and News

**Published:** 1900-02-03

**Authors:** 


### NOTES AND NEWS.

HOSPITAL MEETINGS.

St. John's Hospital for Diseases of the Skin.—Annual Meeting at half-past four p.m., on Friday, February 2nd, at the Westminster Palace Hotel.

St. Mark's Hospital, City Road.—Annual General Meeting at twelve o'clock noon, on Thursday, February 8th, at the Mansion House.

The foundation-stone of a hospital for infectious diseases has been laid at Millport in Scotland by Sir Charles Dalrymple. The institution is to cost £5,000.

An outbreak of diphtheria has occurred at the Merchant Seamen's Orphan Asylum. The drainage is declared to be faulty, and during the process of remedying the defects the children have all been removed.

A Pasteur Institute has been opened at Lyons, and forms the eighth of these institutions in France. In other foreign countries the establishment of Pasteur Institutes continues. In Russia there are at least six, Italy has five, Austria has two, and there is one at Constantinople, Bucarest, Taragossa, Tiflis, Malta, and other places. In America there are about five. In England we have no Pasteur Institute dealing with treatment.

It is stated that Dr. Schenck, the author of a well-known book in which he held that it might be possible by dieting the expectant mother, and by other means, to predetermine the sex of her offspring, has received permission to retire on a pension from the professorship which he held at the University of Vienna. This form of dismissal is said to have been the result of a demand made by the medical faculty. What was the reason given for this demand we do not know, but we are always sorry to find punishment meted out for the holding of opinions, however bizarre they may appear. Now Dr. Schenck undoubtedly offered no proof of his theory, so that its publication was, to say the least of it, premature. Possibly, nay probably, he was wrong in the particular form of treatment which he suggested, but that he was right in his main idea, namely, that the proportion in which male and female children are produced depends in some way upon the nutrition of the parents, is hardly open to doubt.

Dr. Burney Yeo has been appointed Professor Emeritus and Consulting Surgeon to King's College Hospital.

Mr. Thomas Aitkin has given £40,000 to the Edinburgh Royal Infirmary, and in acknowledgment of his munificence the authorities of the infirmary intend to name a ward the Aitkin Ward.

Mr. Robert Gordon has given £500 to clear the deficiency on the accounts of the Dumfries and Galloway Infirmary. Mr. Gordon has given the same sum three years in succession to the infirmary.

Addenbrooke's Hospital, Cambridge, is much to be congratulated, not only upon the munificence of Mr. Peckover, the Lord Lieutenant of the county, towards the institution, but also upon the public spirit which he shows in endeavouring to bring it up to the best modern standard of efficiency.

Dr. A. D. Waller, President-elect of the Neurological Society of London, will deliver an address on "The Excitability of Nervous Matter, with special reference to the Retina," at the annual meeting of the society, on Thursday, February 8th, at half-past eight, in the rooms of the Medical Society, Chandos Street.

Mr. Alfred Mosely, of Hadley Wood, Middlesex, is the latest on the list of the generous private individuals who have offered to provide a hospital for the sick and wounded at the war. Mr. Mosely's offer has been accepted, and he is now organising a hospital for 100 beds, to be stationed near Cape Town. There will be four ward pavilions, to contain 20 patients in each. Mr. Mosely will himself maintain the hospital as long as needful, He is a diamond merchant, of long connection with South Africa.

The Asylums Committee of the London County Council have provided accommodation for about sixty female paying patients having a legal settlement in the county of London at the Manor Asylum, Horton, Epsom. The portion of the building to be utilised for these patients has been specially adapted and fitted up for the purpose. The Epsom railway stations are about one and a half miles distant. The weekly charge is at present fixed at 15s., this being exclusive of clothing and special luxuries. Application is to be made to the Clerk to the Asylums Committee of the London County Council.

We are glad to see that amongst the numerous friends who have come forward to supply comforts for those at the war, the Royal Army Medical Corps has not been forgotten. A committee of influential ladies has been formed amongst whom are the wives of many representative medical men, and of members of the corps. Miss Maxwell Müller, late lady superintendent of the army nursing service, will act as honorary secretary, and will receive subscriptions at 100, Victoria Street, S.W. The corps in South Africa now number 3,000, so that considerable contributions will be required to procure an adequate supply of comforts for the men whose indefatigable and devoted services to the wounded have called forth so much praise.

